# Risk factors analysis of COVID-19 patients with ARDS and prediction based on machine learning

**DOI:** 10.1038/s41598-021-82492-x

**Published:** 2021-02-03

**Authors:** Wan Xu, Nan-Nan Sun, Hai-Nv Gao, Zhi-Yuan Chen, Ya Yang, Bin Ju, Ling-Ling Tang

**Affiliations:** 1 Hangzhou Xiaoshan District Center for Disease Control and Prevention, Hangzhou, China; 2 Hangzhou Wowjoy Information Technology Co., Ltd, Hangzhou, China; 3 State Key Laboratory for Diagnosis and Treatment of Infectious Diseases, National Clinical Research Centre for Infectious Diseases, Collaborative Innovation Centre for Diagnosis and Treatment of Infectious Diseases, the First Affiliated Hospital, College of Medicine, Zhejiang University, Hangzhou, 310003 Zhejiang Province China; 4 Department of Infectious Diseases, ShuLan (Hangzhou) Hospital Affiliated to Zhejiang Shuren University Shulan International Medical College, Hangzhou, China

**Keywords:** Epidemiology, Predictive medicine, Risk factors

## Abstract

COVID-19 is a newly emerging infectious disease, which is generally susceptible to human beings and has caused huge losses to people's health. Acute respiratory distress syndrome (ARDS) is one of the common clinical manifestations of severe COVID-19 and it is also responsible for the current shortage of ventilators worldwide. This study aims to analyze the clinical characteristics of COVID-19 ARDS patients and establish a diagnostic system based on artificial intelligence (AI) method to predict the probability of ARDS in COVID-19 patients. We collected clinical data of 659 COVID-19 patients from 11 regions in China. The clinical characteristics of the ARDS group and no-ARDS group of COVID-19 patients were elaborately compared and both traditional machine learning algorithms and deep learning-based method were used to build the prediction models. Results indicated that the median age of ARDS patients was 56.5 years old, which was significantly older than those with non-ARDS by 7.5 years. Male and patients with BMI > 25 were more likely to develop ARDS. The clinical features of ARDS patients included cough (80.3%), polypnea (59.2%), lung consolidation (53.9%), secondary bacterial infection (30.3%), and comorbidities such as hypertension (48.7%). Abnormal biochemical indicators such as lymphocyte count, CK, NLR, AST, LDH, and CRP were all strongly related to the aggravation of ARDS. Furthermore, through various AI methods for modeling and prediction effect evaluation based on the above risk factors, decision tree achieved the best AUC, accuracy, sensitivity and specificity in identifying the mild patients who were easy to develop ARDS, which undoubtedly helped to deliver proper care and optimize use of limited resources.

## Introduction

The coronavirus disease 2019 (COVID-19) is an acute infectious pneumonia caused by a severe acute respiratory syndrome-coronavirus-2 (SARS-CoV-2) infection previously unknown to humans. Spreading mainly through the droplet route and close contact, the virus causes mild symptoms in the majority of cases, the most common being: fever, dry cough, and fatigue^1,2^.

The disease has the characteristics of fast transmission and strong infectivity^3^. Since the outbreak in early December 2019 in Wuhan, China, it has rapidly developed into a worldwide pandemic, with more than 3 million patients confirmed to have been diagnosed with the disease in more than 200 countries, and the number of infected people is probably much higher. As of April 30, 2020, 217,769 people died of COVID-19 infection. Despite the public health responses aimed at containing the disease and delaying its spread; during the courses of treatment, due to the large increase in the demand for hospital beds and the shortage of medical equipment, coupled with the lack of specific medicine, patients with basic diseases or old age are more likely to progress to severe disease, leading to death. Recent reports show that 14.1–33.0% of COVID-19 infected patients are prone to develop into severe cases, and the mortality rate of critical cases is 61.5%, increasing sharply with age and underlying comorbidities^4–7^. Furthermore, medical staff may also be infected, which makes many countries face critical care crisis. COVID-19 poses an important and urgent threat to global health.

Acute Respiratory Distress Syndrome (ARDS) is a common and devastating critical illness^8^. It has been reported that 67% of COVID-19 patients with the severe illness have developed ARDS, which is the main cause of death^9^. However, in the early stage of onset, quite a few patients have no obvious clinical symptoms, so it is difficult to judge until ARDS occurs. Predicting which patients are more likely to develop ARDS, and thus face a greater risk of complications including death, is particularly important in a novel and accelerating outbreak^10^. It would be useful in evaluation or prediction the public health burden or resources demand in a large scale e.g. in a city or a province.

Artificial intelligence (AI) has begun to tackle these difficult challenges in healthcare and it can provide clinical decision support if used carefully^11^. Currently, the prediction models of COVID-19 reported mainly focus on epidemics trend, early screening, CT diagnosis, and prognosis of COVID-19 patients^12–15^. Few models have been studied for early identification of patients who are most likely to develop ARDS and recommending interventions. Xiang Bai et al. established a Long Short-Term Memory (LSTM) model by combining 75 clinical features and a quantitative CT sequence data obtained at different times to predict the malignant progression of COVID-19, which achieved an AUC of 0.954^16^. Xiangao Jiang et al. used traditional machine learning methods such as decision tree(DT), random forest(RF), and support vector machine(SVM) to predict disease progression to ARDS in COVID-19 patients, with the overall accuracy of 70%-80%^10^. This study was a small sample prediction model of only 53 patients, so the prediction accuracy was slightly lower. The most-reported predictors of severe progression in patients with COVID-19 included age, sex, features derived from computed tomography scans, C reactive protein, lactic dehydrogenase, and lymphocyte count. The C index of these models ranged from 0.85 to 0.98^17^. However, most reports did not include a description of the study population or intended use of the models and were rated at high risk of bias at the same time. Early detection of patients who are likely to develop critical illness is of great importance and may help to deliver proper care and optimize use of limited resources. We aimed to develop the COVID-19 ARDS clinical decision support system using machine learning algorithms and deploy it into electronic medical records(EMR) to assist doctors in identifying severe patients at the time of hospital admission.

## Results

### Characteristics of COVID-19 patients

Tables 1, 2 and 3 lists the distribution of various parameters including demographic, epidemiology and clinical characteristics of the COVID-19 ARDS and non-ARDS populations.

**Table 1:** Demographic and epidemiology of the study patients.

Characteristics	Patients (N = 659)	ARDS patients (N = 76)	Non-ARDS patients (N = 583)	P value
**Demography**				
Age				
Median(IQR)- years	50.0 (37.0–62.0)	56.5(47.5–67.8)	49.0(36.0–60.0)	0.000
Distribution—no./total no. (%)				0.000
14–30 years	70/659(10.6%)	2/76(2.6%)	68/583(11.7%)	
30–70 years	545/659(82.7%)	61/76(80.3%)	484/583(83%)	
≥ 70 years	44/659(6.7%)	13/76(17.1%)	31/583(5.3%)	
Gender				0.000
Male—no./total no. (%)	332/659(50.4%)	58/76(76.3%)	274/583(47.0%)	
Female—no./total no. (%)	327/659(49.6%)	18/76(23.7%)	309/583(53.0%)	
BMI				
Median(IQR)	23.9(21.5–25.9)	25.5(21.5–25.7)	23.7(23.2–27.7)	0.001
Distribution—no./total no. (%)				0.007
< 23	229/591(38.7%)	14/65(21.5%)	215/526(40.9%)	
23–25	161/591(27.2%)	17/65(26.2%)	144/526(27.4%)	
> 25	201/591(34.1%)	34/65(52.3%)	167/526(31.7%)	
Medical staff—no./total no. (%)	9/659(1.4%)	1/76(1.3%)	8/583(1.4%)	0.968
Pregnancy history—no./total no. (%)	11/659(1.67%)	3/76(3.9%)	8/583(1.4%)	0.123
Smoking history—no./total no. (%)	51/654(7.8%)	11/76(14.5%)	40/578(6.9%)	0.198
**Epidemiology—no./total no. (%)**				
History of exposure	447/630(70.9%)	38/70(54.3%)	409/560(73.0%)	0.002
Family members have disease	258/583(44.3%)	27/76(35.5%)	231/507(45.6%)	0.285
Number of cases in family members—Median(IQR)	1(0–13)	1(0–1)	1(0–1)	0.263
Have been to Wuhan	381/657(57.9%)	34/76(44.7%)	347/581(59.7%)	0.091
Stay in ICU	33/608(5.4%)	33/73(45.2%)	0/535(0%)	0.000
Interval between date of contact and date of onset—Median (IQR)	5(3–9)	6(3–8)	5(3–10)	0.901
The interval between the onset date and the visit date				
Median (IQR)	2(0–5)	3(1–6)	1(0–4)	0.772
Distribution—no./total no. (%)				0.035
≤ 2 days	385/651(59.1%)	34/75(45.3%)	351/576(60.9%)	
3–5 days	142/651(21.8%)	22/75(29.3%)	120/576(20.8%)	
> 5 days	124/651(19.0%)	19/75(25.3%)	105/576(18.3%)	
The interval between the date of onset and the date of admission				
Median (IQR)	5(2–10)	6(3–8)	5(3–8)	0.759
Distribution—no./total no. (%)				0.250
≤ 2 days	182/656(27.7%)	15/76(19.7%)	167/580(28.8%)	
3–5 days	167/656(25.5%)	22/76(28.9%)	145/580(25.0%)	
> 5 days	307/656(46.8%)	39/76(51.3%)	268/580(46.2%)	
The interval between the onset date and the antiviral date				
Median (IQR)	5(3–10)	4(4–9)	5(4–9)	0.278
Distribution—no./total no. (%)				0.250
≤ 2 days	182/656(27.7%)	15/76(19.7%)	167/580(28.8%)	
3–5 days	167/656(25.5%)	22/76(28.9%)	145/580(25.0%)	
> 5 days	307/656(46.8%)	39/76(51.4%)	268/580(46.2%)	

BMI, body mass index; ICU, intensive care unit; no., number.

Data are presented as medians (interquartile ranges, IQR) and no./total no. (%).

**Table 2:** clinical characteristics and underlying diseases.

Characteristics	Patients (N = 659)	ARDS patients (N = 76)	Non-ARDS patients (N = 583)	P value
**Clinical symptoms—no./total no. (%)**				
Severity evaluation at admission				0.000
Mild	59/643(9.2%)	0/76(0.0%)	59/567(10.4%)	
Ordinary	485/643(75.4%)	15/76(19.7%)	470/567(82.9%)	
Severe	67/643(10.4%)	29/76(38.2%)	38/567(6.7%)	
Critical	32/643(5.0%)	32/76(42.1%)	0/567(0.0%)	
Fever on the first day of admission—temperature	439/659(66.6%)	65/76(85.5%)	374/583(64.2%)	0.000
Median (IQR)—℃	37.5(36.8–38.2)	37.9(37.3–38.5)	37.4(36.8–38.1)	0.000
Distribution—no./total no. (%)				0.000
< 37.3℃	275/659(41.7%)	18/76(23.7%)	257/583(44.1%)	
37.3–38℃	198/659(30.0%)	24/76(31.6%)	174/583(29.8%)	
38.1–39℃	153/659(23.2%)	24/76(31.6%)	129/583(22.1%)	
> 39℃	33/659(5.0%)	10/76(13.2%)	23/583(3.9%)	
Cough	452/658(68.7%)	61/76(80.3%)	391/582(67.2%)	0.025
Expectoration	261/659(39.6%)	37/76(48.7%)	224/583(38.4%)	0.105
Dry cough	191/644(29.7%)	26/75(34.7%)	165/569(29.0%)	0.495
Yellow sputum	32/643(5.0%)	3/73(4.1%)	29/570(5.1%)	0.049
Hemoptysis	10/626(1.6%)	4/73(5.5%)	6/553(1.1%)	0.017
Sore throat	81/635(12.8%)	9/72(12.5%)	72/563(12.8%)	0.051
Stuffy nose	24/631(3.8%)	1/72(1.4%)	23/559(4.1%)	0.506
Muscle ache	108/633(17.1%)	7/72(9.7%)	101/561(18.0%)	0.095
Fatigue	221/646(34.2%)	27/73(36.9%)	194/573(33.9%)	0.366
Shortness of breath	110/635(17.3%)	45/76(59.2%)	65/559(11.6%)	0.000
Gastrointestinal symptoms	121/645(18.8%)	14/74(18.9%)	107/571(18.7%)	0.948
Diarrhea	91/607(15.0%)	15/73(20.5%)	76/534(14.2%)	0.179
Vomiting	18/603(3.0%)	5/73(6.8%)	13/530(2.4%)	0.054
Nausea	60/609(9.9%)	5/73(6.8%)	55/536(10.3%)	0.323
Encephalopathy	3/617(0.5%)	0/73(0.0%)	3/544(0.6%)	0.590
Headache	67/614(10.9%)	6/73(8.2%)	61/541(11.3%)	0.450
**Underlying diseases—no./total no. (%)**				
Basic disease	274/657(41.7%)	43/76(56.6%)	231/581(39.8%)	0.006
Hypertension	167/641(26.1%)	37/76(48.7%)	130/565(23.0%)	0.000
Heart disease	23/628(3.7%)	3/73(4.1%)	20/555(3.6%)	0.829
Diabetes	66/628(10.5%)	13/73(17.8%)	53/555(9.5%)	0.041
Fatty liver	65/610(10.7%)	12/73(16.4%)	53/537(9.9%)	0.088
COPD	7/614(1.1%)	1/73(1.4%)	6/541(1.1%)	0.590
Asthma	3/614(0.5%)	0/73(0.0%)	3/541(0.6%)	0.524
Malignancy	9/629(1.4%)	1/73(1.4%)	8/556(1.4%)	0.971
Immunosuppressant	1/614(0.2%)	1/73(1.4%)	0/541(0.0%)	0.119
Blood system diseases	2/613(0.3%)	1/73(1.4%)	1/540(0.2%)	0.096
Chronic hepatopath	29/631(4.6%)	5/73(6.8%)	24/558(4.3%)	0.328
Chronic nephrosis	6/616(1.0%)	1/73(1.4%)	5/543(0.9%)	0.756
Pneumonia on admission	591/647(91.3%)	75/76(98.7%)	516/571(90.4%)	0.056
Acute kidney injury on admission	9/652(1.4%)	3/76(3.9%)	6/576(1.0%)	0.076
With other respiratory virus infections	5/569(0.9%)	2/73(2.7%)	3/496(0.6%)	0.126

COPD, chronic obstructive pulmonary disease; no., number.

Data are presented as medians (interquartile ranges, IQR) and no./total no. (%).

**Table 3:** Radiologic, laboratory findings and complications.

Variables	Patients (N = 659)	ARDS patients (N = 76)	Non-ARDS patients (N = 583)	P value
**CT image features—no./total no. (%)**				
Consolidation	175/619(28.3%)	41/76(53.9%)	134/543(24.7%)	0.000
Ground-glass opacity	467/625(74.7%)	59/73(80.8%)	408/552(73.9%)	0.251
Number of consolidation quadrant—Median (IQR)	0(0–2)	2(1–4)	0(0–2)	0.000
**Laboratory findings**				
Leukocyte count within 48 h of admission (10e9/l)				
Median (IQR)	5.0(4.0–6.6)	5.7(4.6–8.8)	4.9(3.9–6.4)	0.000
Distribution—no./total no. (%)				0.000
< 4	163/657(24.8%)	14/76(18.4%)	149/581(25.6%)	
4–10	468/657(71.2%)	47/76(61.8%)	421/581(72.5%)	
> 10	26/657(4.0%)	15/76(19.7%)	11/581(1.9%)	
Neutrophil count (10e9/l) -Median(IQR)	3.2(2.3–4.4)	4.8(3.1–7.6)	3.1(2.3–4.2)	0.000
Lymphocyte count (10e9/l)				
Median (IQR)	1.1(0.8–1.6)	0.7(0.5–1.1)	1.2(0.8–1.7)	0.000
Distribution—no./total no. (%)				0.000
≤ 1.5	468/659(71.0%)	71/76(93.4%)	397/583(68.1%)	
> 1.5	191/659(29.0%)	5/76(6.6%)	186/583(31.9%)	
Lymph%				
Median (IQR)	0.24(0.2–0.3)	0.13(0.1–0.2)	0.25(0.2–0.3)	0.403
Distribution—no./total no. (%)				
< 0.2	237/657(36.1%)	56/76(73.7%)	181/581(31.2%)	0.000
≥ 0.2	420/657(63.9%)	20/76(26.3%)	400/581(68.8%)	
Neutrophils / lymphocytes				
Median (IQR)	2.73(1.8–4.5)	6.11(3.7–14.6)	1.2(1.7–4.0)	0.000
Distribution—no./total no. (%)				0.000
< 3	348/636(54.7%)	13/75(17.3%)	335/561(59.7%)	
≥ 3	288/636(45.3%)	62/75(82.7%)	226/561(40.3%)	
Hemoglobin (g/l)- Median (IQR)	134.0(123.0–146.0)	135.5(123.3–150.0)	134(123.0–144.0)	0.462
Hematocrit (%)- Median (IQR)	0.4(0.0–0.4)	0.4(0.3–0.4)	0.39(0.4–0.5)	0.968
Platelet (10e9/l)				
Median (IQR)	195(152–243)	174(138–222)	197(154–243)	0.012
Distribution—no./total no. (%)				0.509
≤ 100	24/658(3.6%)	4/76(5.3%)	20/582(3.4%)	
> 100	634/658(96.4%)	72/76(94.7%)	562/582(96.6%)	
Alanine aminotransferase (U/L)				
Median (IQR)	21.0(15–34.0)	30.3(21.3–46.0)	20.0(14.0–32.0)	0.000
Distribution—no./total no. (%)				0.000
≤ 40	533/654(81.5%)	48/74(64.9%)	485/580(83.6%)	
> 40	121/654(18.5%)	26/74(35.1%)	95/580(16.4%)	
Aspartate aminotransferase (U/L)				
Median (IQR)	24.0(18.0–31.0)	31.5(24.0–46.0)	23(18.0–30.0)	0.000
Distribution—no./total no. (%)				0.000
≤ 40	563/653(86.2%)	44/74(59.5%)	519/579(89.6%)	
> 40	90/653(13.8%)	30/74(40.5%)	60/579(10.4%)	
Potassium ion (mmol/L)—median(IQR)	3.8(3.6–4.2)	3.8(3.5–4.1)	3.9(3.6–4.2)	0.188
Sodium ion (mmol/L)—median(IQR)	138.8(136.7–146.7)	138.0(135.0–140.6)	139.0(137.0–140.7)	0.052
Creatinine (umol/l)				
Median (IQR)	65.3(54.0–78.0)	70.5(59.5–92.0)	65.0(53.4–77.4)	0.002
Distribution—no./total no. (%)				0.072
≤ 133	648/657(98.6%)	72/75(96.0%)	576/582(99.0%)	
> 133	9/657(1.4%)	3/75(4.0%)	6/582(1.0%)	
Creatine Kinase (U/L)				
Median (IQR)	74.0(50.8–107.2)	112.5(63.0–245.0)	70.6(49.0–96.3)	0.000
Distribution—no./total no. (%)				0.000
≤ 185	461/513(89.9%)	47/68(69.1%)	414/445(93.0%)	
> 185	52/513(10.1%)	21/68(30.9%)	31/445(7.0%)	
Lactate dehydrogenase (U/L)				
Median (IQR)	215.0(177.0–275.3)	316.0(253.8–394.0)	211.0(173.5–257.5)	0.000
Distribution—no./total no. (%)				0.000
≤ 250	431/636(67.8%)	17/69(24.6%)	414/567(73.0%)	
> 250	205/636(32.2%)	52/69(75.4%)	153/567(27.0%)	
Troponin I (ng/ml—Median (IQR)	0.0(0.01–0.06)	0.0(0.00–0.03)	0.0(0.01–0.09)	0.013
Brain Natriuretic Peptide (< 300 pg/ml)—Median (IQR)	82.0(27–144.5)	77.0(36.0–164.0)	82.3(26.5–139.7)	0.566
Myoglobin—Median (IQR)	25.1(18.5–40.6)	85.9(43.2–205.9)	23.6(18.2–33.1)	0.000
Glucose (mmol/L)—Median (IQR)	5.9(5.1–7.7)	8.1(6.6–9.9)	5.7(5.1–7.4)	0.000
C-reactive protein (mg/L)				
Median (IQR)	10(3.0–27.3)	34.1(15.3–76.0)	8.8(2.8–24.1)	0.000
Distribution—no./total no. (%)				0.000
≤ 10	341/648(52.6%)	14/73(19.2%)	327/575(56.9%)	
> 10	307/648(47.4%)	59/73(80.8%)	248/575(43.1%)	
Procalcitonin (ng/L)				
Median (IQR)	0.1(0.0–0.1)	0.1(0.1–0.2)	0.1(0.0–0.1)	0.008
Distribution—no./total no. (%)				0.195
< 0.1	326/510(63.9%)	37/68(54.4%)	289/442(65.4%)	
0.1–0.5	164/510(32.2%)	27/68(39.7%)	137/442(31.0%)	
≥ 0.5	20/510(3.9%)	4/68(5.9%)	16/442(3.6%)	
Highest CRP within one week of admission (mg/L)—Median (IQR)	15.5(4.0–39.6)	45.8(17.7–83.1)	12.2(3.8–34.2)	0.000
Highest PCT within one week of admission (ng/L)—Median (IQR)	0.1(0.0–0.1)	0.1(0.1–0.2)	0.1(0.0–0.1)	0.000
FiO2 on the first day of admission—Median (IQR)	0.3(0.2–0.3)	0.2(0.3–0.5)	0.3(0.2–0.3)	0.000
PaO2 on the first day of admission (mmHg)—Median (IQR)	90.7(78.0–111.0)	79.0(67.9–95.2)	94.0(80.2–117.0)	0.019
PaCO2 on the first day of admission (mmHg)—Median (IQR)	37.5(34.0–41.5)	33.0(32.4–37.5)	38.0(34.8–41.9)	0.699
Oxygenation index on the first day of admission (mmHg)				
Median (IQR)	292.2(144.4–424.3)	240(160.8–261.2)	352.0(64.2–466.2)	0.000
Distribution—no./total no. (%)				0.000
≤ 200	106/325(32.6%)	35/69(50.7%)	71/256(27.7%)	
> 200	219/325(67.4%)	34/69(49.3%)	185/256(72.3%)	
D-dimer (μg/L)—Median (IQR)	250.0(140.0–525.3)	819.0(276.0–1212.0)	238.0(130.0–458.0)	0.000
**Complications—no./total no. (%)**				
Shock	4/647(0.6%)	4/73(5.5%)	0/574(0.0%)	0.000
Secondary bacterial infection	48/659(7.3%)	23/76(30.3%)	25/583(4.3%)	0.000

CT, computerized tomography; CRP, c-reactive protein; PCT, procalcitonin; no., number.

Data are presented as medians (interquartile ranges, IQR) and no./total no. (%).

#### Demographics and epidemiology

In this study, we collected a total of 659 patients from Wuhan and non-Wuhan areas who were confirmed with COVID-19, of which 76 patients (11.5%) developed ARDS. 447 patients (70.9%) had contact with infected persons and 44.3% had a family infection. The median incubation period was 5 days (interquartile range, 3 to 9) and the average time from onset to ARDS and admission to ARDS were 10 days and 3 days, respectively. The median age of the patients was 50 years (interquartile range, 37 to 62) and 50.4% of the patients were male. Patients with ARDS were significantly older than those with non-ARDS by a median of 7.5 years (56.5 years vs. 49 years) and male patients (76.3%) were more likely to develop ARDS. More than 50% of ARDS patients had a BMI greater than 25. However, the exposure histories of the two groups were similar (Table 1).

#### Clinical characteristics and underlying diseases

On severity evaluation at admission, 75.4% of COVID-19 patients were assessed as ordinary type while among the patients with ARDS, 80.3% were evaluated as severe or critical. The most common clinical symptoms of COVID-19 patients at the time of onset were fever (66.6%), cough (68.7%), expectoration (39.6%), fatigue (34.2%) and dry cough (29.7%). Encephalopathy (0.5%), hemoptysis (1.6%), vomiting (3.0%) and stuffy nose (3.8%) were uncommon. Compared with non-ARDS patients, ARDS patients had a higher frequency of coughing (80.3% vs. 67.2%) and dyspnea (59.2% vs. 11.6%). The median temperature was 37.5°C. ARDS patients were 0.5°C higher than non-ARDS patients (37.9℃ vs. 37.4℃), which was statistically significant (P < 0.001).

Overall, the presence of any comorbidities was more common among ARDS patients than no-ARDS (56.6% vs. 39.8%). Patients with ARDS had a much higher incidence of hypertension (48.7% vs.23%) and diabetes (17.8% vs.9.5%). Two of the five patients infected with other viruses developed ARDS. ARDS also occurred in one patient who was treated with immunosuppressive agents (Table 2).

#### Radiologic, laboratory findings and complications

Table 3 shows the results of radiologic, laboratory findings on admission and complications. 74.7% of the patients presented ground-glass shadows on chest CT images and 28.3% of the patients presented consolidation. The above two imaging features accounted for a higher proportion of patients with ARDS than non-ARDS patients, which were 80.8% vs 73.9% and 53.9% vs 24.7%, respectively. The median number of consolidation quadrant in ARDS patients was two.

Within 48 h of admission, lymphocytopenia was present in 36.1% of the patients and leukopenia in 24.8%. However, among ARDS patients, 19.7% had an increase in the white blood cell count, which indicated that ARDS patients had a secondary infection. The ratio of neutrophils to lymphocytes was greater than 3 in 45.3% of COVID-19 patients and 82.7% in ARDS patients with a median of 6.11. 47.4% and 32.2% of patients had elevated levels of C-reactive protein and lactate dehydrogenase, respectively. In a small number of patients, levels of alanine aminotransferase (ALT), glutamate aminotransferase (AST), creatine kinase (CK) and D-dimer were elevated. Laboratory abnormalities were more severe in ARDS patients than in non-ARDS patients. Besides, the medians of myoglobin and fasting glucose in ARDS patients were 85.9 μg/L and 8.1 mmol/L respectively, which exceeded the normal reference range and was significantly different from the non-ARDS group.

During hospitalization, 91.3% of patients were diagnosed with pneumonia, and there was no statistical difference between the ARDS group and non-ARDS group. However, patients with ARDS had a higher incidence of shock and secondary bacterial infection (5.5% and 30.3%) than those with non- ARDS (0 and 4.3%), and 45.2% of them were admitted to ICU (Tables 2, 3).

### Prediction of risk factors for COVID-19 ARDS

After removal of variables with missing rate > 20%, a total of 98 variables consisting of demographic, epidemiology, clinical symptoms, underlying diseases, complication, CT image features and laboratory results were extracted from the structured and unstructured data of electronic medical record (EMR) according to literature reviews and expert clinician opinions. Then, we selected 19 significant risk factors related to COVID-19 by means of SPSS single factor analysis. Among all risk factors, severity evaluation at admission (odds ratio [OR], 13.206; 95%CI, 8.550–20.397; P < 0.001), gender (OR, 3.312; 95%CI, 1.979–5.544; P < 0.001), age (≥ 70 year) (OR, 19.811; 95%CI, 4.473–87.741; P < 0.001), BMI (< 23 vs. > 25) (OR, 3.717; 95%CI, 1.966 -7.062; P < 0.001), temperature (> 39℃) (OR, 5.279; 95%CI, 2.305–12.090; P < 0.001), hemoptysis (OR, 7.307; 95%CI, 2.263–23.595; P < 0.001), cough (OR, 2.574; 95%CI, 1.429–4.542; P < 0.001), shortness of breath (OR, 11.281; 95%CI, 6.883–18.490; P < 0.001), hypertension (OR, 4.105; 95%CI, 2.572–6.554; P < 0.001), diabetes (OR, 2.176; 95%CI, 1.161–4.078; P < 0.001), secondary bacterial infection (OR, 9.686; 95%CI, 5.146–18.323; P < 0.001), lung consolidation (OR, 4.264; 95%CI, 2.668–6.815; P < 0.001), lymphocyte count (OR, 0.145; 95%CI, 0.080–0.263; P < 0.001), neutrophils/lymphocytes ratio (NLR) (< 3 vs. ≥ 3) (OR, 7.211; 95%CI, 3.980–13.064; P < 0.001), ALT(≤ 40 vs. > 40 U/L) (OR, 2.710; 95%CI, 1.639–4.482; P < 0.001), AST (≤ 40 vs. > 40 U/L) (OR, 5.139; 95%CI, 3.100–8.520; P < 0.001), CK (≤ 185 vs. > 185 U/L) (OR, 4.114; 95%CI, 2.312–7.319; P < 0.001), lactate dehydrogenase (LDH) (≤ 250 vs. > 250 U/L) (OR, 8.104; 95%CI, 4.733–13.876; P < 0.001), C-reactive protein (CRP) (≤ 10 vs. > 10 mg/L) (OR, 5.959; 95%CI, 3.510–10.119; P < 0.001) were all strongly correlated with ARDS (Table 4).

**Table 4:** Risk factor analysis for COVID -19.

Characteristics	OR	95% CI	P value
Gender (male vs. female)	3.312	1.979–5.544	0.000
Age ≥ 70 years	19.811	4.473–87.741	0.001
BMI > 25	3.717	1.966 -7.062	0.001
Severity evaluation at admission	13.206	8.550–20.397	0.000
Temperature > 39 °C	5.279	2.305–12.090	0.000
Cough	2.574	1.429–4.542	0.002
Shortness of breath	11.281	6.883–18.490	0.000
Hemoptysis	7.307	2.263–23.595	0.000
Hypertension	4.105	2.572–6.554	0.000
Diabetes	2.176	1.161–4.078	0.015
Secondary bacterial infection	9.686	5.146–18.323	0.000
Lung consolidation	4.264	2.668–6.815	0.000
Lymphocyte count (10e9/l)	0.145	0.080–0.263	0.000
Neutrophils/lymphocytes (< 3 vs. ≥ 3)	7.211	3.980–13.064	0.000
Alanine aminotransferase (≤ 40 vs. > 40 U/L)	2.710	1.639–4.482	0.000
Aspartate aminotransferase (≤ 40 vs. > 40 U/L)	5.139	3.100–8.520	0.000
Creatine kinase (≤ 185 vs. > 185 U/L)	4.114	2.312–7.319	0.000
Lactate dehydrogenase (≤ 250 vs. > 250 U/L)	8.104	4.733–13.876	0.000
C-reactive protein (≤ 10 vs. > 10 mg/L)	5.959	3.510–10.119	0.000

### Development and verification of predictive models

**Figure 1:**
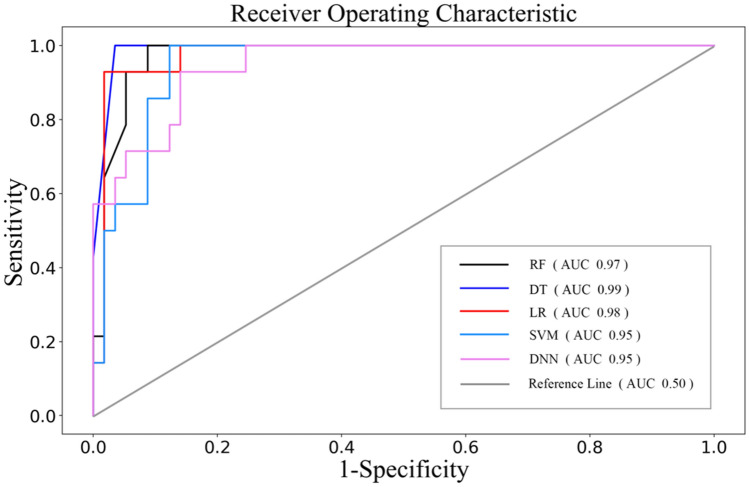
ROC of the five algorithms on the external testing dataset.

Based on the above results of univariate analysis, we determined 19 risk factors including severity evaluation at admission, gender, age, BMI, temperature, cough, shortness of breath, hemoptysis, hypertension, diabetes, secondary bacterial infection, lung consolidation, lymphocyte count, CK, NLR, ALT, AST, LDH, and CRP as inputs to the model to evaluate whether COVID-19 patients would develop ARDS. We tried five algorithms for modeling, including logistic regression (LR), random forest (RF), support vector machine (SVM), decision tree (DT) and deep neural networks (DNN). Table 5 shows the mean ± standard deviation (std.) for 10-fold cross validation with AUC and accuracy. DT, LR and RF all exceeded AUC of 0.85 and the mean accuracy of each algorithm was over 0.8. In order to further verify the accuracy of the models, performances of five algorithms were evaluated on the external test set with each technique. Table 6 and Fig. 1 show that DT, LR, RF and DNN all demonstrated good performance in term of AUC, accuracy and specificity. The sensitivity of DT and LR was much higher than that of other three models. Considering the unbalance of the actual dataset, we also evaluated the balanced accuracy of each model. The result of DT and DNN was 0.98 and 0.93, respectively. The predictive model established by SVM exhibited the worst performance in five models. It is necessary for ARDS diagnosed tool with high sensitivity and accuracy. The results show that DT marked the best value in each evaluation with AUC of 0.99, accuracy of 0.97 and sensitivity of 1.0 respectively. Therefore, the model constructed by decision tree algorithm was optimum tool for ARDS prediction.

**Table 5:** 10-fold cross-validation results of 5 algorithms.

Models	AUC (mean ± std)	Accuracy (mean ± std)
Decision tree	0.89 ± 0.06	0.88 ± 0.06
Logistic regression	0.86** ± **0.07	0.87 ± 0.06
Random forest	0.86** ± **0.06	0.85 ± 0.06
Support vector machine	0.83** ± **0.08	0.81 ± 0.05
Deep neural networks	0.74** ± **0.12	0.88 ± 0.06
P-value	0.002	0.050

**Table 6:** Performance of the five algorithms on external testing dataset in predicting the occurrence of COVID-19 ARDS.

Models	AUC	Accuracy	Sensitivity	Specificity	F-measure (no-ARDS/ARDS)	Balance accuracy
Decision tree	0.99	0.97	1.00	0.96	0.98/0.93	0.98
Logistic regression	0.98	0.93	0.93	0.93	0.95/0.84	0.87
Random forest	0.97	0.92	0.79	0.95	0.95/0.79	0.83
Support vector machine	0.95	0.83	0.14	1.00	0.90/0.25	0.57
Deep neural networks	0.95	0.90	0.71	0.95	0.94/0.74	0.93

## Discussion

In this study, we comprehensively compared the clinical characteristics of all confirmed COVID-19 patients with and without ARDS, and determined 19 features for modeling. All included variables were strongly correlated with disease progression. Age (> 70 years), gender, hypertension, diabetes as well as severity evaluation are recognized risk factor for developing ARDS in COVID-19 patients^18^. Clinical manifestations such as fever, cough, hemoptysis, shortness of breath and lung consolidation reflect the progression of COVID-19^19,20^. Viral infections predispose patients to secondary bacterial infections, which often lead to a more severe clinical course. Secondary bacterial infection has been considered as a critical risk factor for the severity and mortality rates of COVID-19 despite antimicrobial therapies^21,22^. Lymphopenia, high concentrations of CRP and LDH may indicate severe acute lung inflammatory reaction and cell damage^23–25^, which has been reported to be risk factors for severe patients with COVID-19^26^. ALT and AST are markers of acute liver injury. Studies have found that abnormal liver tests in patients with COVID-19 were associated with the progression to severe pneumonia. The detrimental effects on liver were mainly related to the use of lopinavir/ritonavir during hospitalization. Therefore, liver function should be monitored and evaluated frequently during medication^27,28^. NLR is an indicator of systemic inflammation^29^, mainly seen in tumor-related diseases, autoimmune diseases, bacterial infectious pneumonia and tuberculosis^30–33^. It was reported that COVID-19 infection-triggered inflammation increased NLR, which was significantly associated with poor clinical outcomes of COVID-19 patients^34^. We found that CK was a high-risk factor for ARDS. On the one hand, it might be associated with heart injury in critically ill patients with COVID-19^35^. On the other hand, this indicator was related to rhabdomyolysis^36,37^. Several cases of rhabdomyolysis were reported in COVID-19 severe patients, with a marked increase of CK^38–40^.

We tried five algorithms for modeling and finally the decision trees performed best. In clinical prediction research, decision tree is frequently designed to build binary classifiers, such as cancer prediction/prognosis^41^. As a method used in machine learning, it is nonparametric which makes fewer data assumptions and it can accommodate collinear independent variables^42^. It is also less sensitive to outliers and more robust to high-dimensional data, which possess many independent variables relative to outcomes^43^. The main advantage of decision tree is its simple structure, which allows for better extracting classification rules and interpretation. Our model consisted of 19 clinical variables, which were all relatively inexpensive and easy to be obtained directly from clinical symptoms and routine laboratory tests. At the same time, the system showed good sensitivity, specificity and AUC in the external test cohort. Compared with the results of Jiang et al.^10^, the overall accuracy of our model is higher (70% vs 91%).

Our study has several strengths: first, we have successfully used a machine learning algorithm to analyze clinical datasets and developed a diagnosis aid system, which has been deployed in electronic medical records for early identification of ARDS in COVID-19 patients. By submitting clinical information online, medical staff can triage patients at hospital admission based on the predicted risk factors and arrange patient treatment plans accordingly, ensuring patients receive treatment early and medical resources can be efficiently allocated. Secondly, to ensure the reliability of the conclusion, we used data from multi-centers with large samples for modeling and verification. Third, we found that CK (> 185 U/L) and NLR were strongly correlated with ARDS, which might be the new potential early identification biomarkers in COVID-19 severe patients.

There are still some deficiencies in our study and we have a lot of works to do in the future. Firstly, although we collected data of 659 COVID-19 patients in multiple centers, samples available for ARDS were limited. Secondly, we did not collect CT images data, and the quantitative information of CT diagnostic data was not detailed enough. Thirdly, it has been reported that D-dimer was a risk factor for COVID-19 severity. However, due to a large number of missing data, similar conclusions were not reached in our study. Finally, it is of great clinical value to study the intervention measures and prognosis of COVID-19 patients before and after the development of ARDS and integrate them into the diagnostic system to achieve personalized recommendations of treatment measures.

## Conclusion

We retrospectively analyzed the clinical characteristics of COVD-19 patients with and without ARDS from Zhejiang Province and Wuhan and identified 19 risk factors. Further, based on these risk factors, we used five methods for modeling, four of which had good predicting effect. The decision tree performed best with an accuracy rate of 97%. We have deployed it to the infectious disease electronic medical record system to assist doctors in early warning severe patients with COVID-19.

## Method

### Patient population and clinical data

Data on a total of 659 consecutive COVID-19 patients from January 22 to April 1, 2020 were retrospectively collected in hospitals from 11 regions: NingBo, ZhouShan, HuBei, Lishui, Jiaxin, HangZhou, TaiZhou, DongYang, ShaoXing, WenZhou, HuZhou. The age of the patient ranged from 14 and 90 years old. All patients were diagnosed by positive tests of severe acute respiratory syndrome-coronavirus-2(SARS-CoV-2) nucleic acids, according to WHO interim guidance. Clinical information including demographic, comorbidities, epidemiological history of exposure to COVID-19, vital sign, clinical symptoms, biochemical indices, blood routine, infection-related biomarkers, CT findings, therapeutic measures, and all the time information from onset to admission were collected from routine clinical practice. The date of disease onset was defined as the day when symptoms (i.e. fever, dry cough, expectoration, polypnea, fatigue, myalgia, pharyngalgia, dyspnea, headache, vomiting) first appeared. ARDS was defined according to the Berlin definition. Severity evaluation criteria on admission was based on the *Guidelines for the Diagnosis and Treatment of Novel Coronavirus (2019-nCoV) Infection (Trial Version 7)*, which was a comprehensive evaluation index with important clinical diagnostic value. Patients with one of the following symptoms are diagnosed as secondary bacterial infection: bacteria are found in sterile sites; patients have a fever that was unrelated to the initial disease, accompanied by elevated CRP. This study was approved by the Ethics Committee of Shulan Hangzhou Hospital. Written informed consents were signed during hospitalization from patients or their parents.

### Data analysis

Continuous variables were expressed as medians and interquartile ranges or simple ranges, as defined by experts. Categorical variables were summarized as counts and percentages. We assessed differences between ARDS and non-ARDS using Two-Sample T test or Mann–Whitney U test depending on parametric or non-parametric data for continuous variables and the Chi-square for categorical variables. Tests were two-sided with significance set at α less than 0·05. All statistical analysis was performed using IBM SPSS Ver. 19.0. The Python programming language (Python Software Foundation, version 3.6.6, https://www.python.org/downloads/) was used for our models.

### Machine learning model establishment and evaluation

#### Datasets

All data was divided into three separate parts with no overlapping topics: training, validation, and external test sets (Table 7).

**Table 7:** Details of modeling datasets.

Datasets	Patients	ARDS patients	No-ARDS patients
Training and validation dataset	236	47	189
External test dataset	71	14	57

For COVID-19 ARDS prediction.

- Training and validation datasets: 236 subjects were assigned to the training and validation datasets following a 9:1 ratio, including 189 non-ARDS and 47 ARDS cases from 11 regions in Wuhan and Zhejiang, further cross-validated 10 times. These datasets were used to train model parameters.
- External test dataset: There were 57 non-ARDS and 14 ARDS cases from 11 regions in Wuhan and Zhejiang. This dataset was used to evaluate and analyze the performances of different models to select the best model for AI system.

#### Algorithms

Four conventional types of machine learning algorithms (decision trees, random forests, support vector machines and logistic regression) and one deep learning method with ReLu activation function (deep neural networks, DNN) were conducted to develop the ARDS prediction model in COVID-19 patients. We implemented support vector machines with the RBF kernel. ID3 decision tree was constructed with the max leaf nodes of 5 and random forest was constructed by 40 decision trees with criterion of entropy algorithm. The pipeline of the DNN model was shown in Figure S1. The input data was a 19-dimensional vector, containing the clinical data of patients. The DNN model employed in this study was a 4-layer network structure with the hidden neurons of 64, 32, 8 and 1 respectively. A sigmoid layer was added at the top of the network to output the probability of ARDS occurrence and a total of 100 epochs were executed.

#### Evaluation

The performance of the models was assessed by 10-fold cross validation (10-fold CV) and external tests. Specifically, we randomly divided the training and validation datasets into 10 parts: 9 parts were used to train the algorithms and 1 part was used to estimate the prediction performance of the method. The mean AUC and accuracy were calculated by 10-fold CV as indicators of prediction accuracy. This process was repeated 10 times. Furthermore, we verify the prediction accuracy of the models on the external test dataset by evaluating the receiver operating characteristic (ROC) curves, the classification accuracy, F-measure, sensitivity and specificity.

### Application development

The best algorithm for ARDS risk prediction was embedded into EMR and could be accessed via the link https://ai-ards.rubikstack.com/#/login. The Anaconda Distribution (Anaconda Inc, Austin, Texas), Visual Studio Code version 1.45.1 (Microsoft, Redmond, Washington), and Python version 3.6 (Python Software Foundation, Wilmington, Delaware) were used for data analysis, model creation, and web application development.

### Ethics approval and consent to participate

This study has been approved by the ethics committee of ShuLan (Hangzhou) Hospital. All procedures performed in studies involving human participants were in accordance with the ethical standards of the institutional and/or national research committee and with the 1964 Helsinki declaration and its later amendments or comparable ethical standards. Written informed consents were signed during hospitalization from patients or their parents. The data used in this study were anonymized before its use.

## Supplementary Information

Supplementary Information 1.

Supplementary Information 2.

Supplementary Information 3.
