# Supplementary material for: Risk factors analysis of COVID-19 patients with ARDS and prediction based on machine learning
Source: Sci Rep. 2021 Feb 3;11:2933. doi: 10.1038/s41598-021-82492-x (PMC7858607; doi:10.1038/s41598-021-82492-x)
Supplement: Supplementary file 1 — Supplementary Information 1. [file 41598_2021_82492_MOESM1_ESM.docx]

# import os
# os.environ['KERAS_BACKEND']='tensorflow'
import pandas as pd
from sklearn.preprocessing import MinMaxScaler,StandardScaler
from sklearn.model_selection import train_test_split,cross_val_score,GridSearchCV, KFold
from sklearn.linear_model import LogisticRegression
from sklearn.pipeline import Pipeline
from sklearn import metrics
from sklearn.metrics import mean_absolute_error, accuracy_score,confusion_matrix,classification_report
from sklearn.tree import DecisionTreeClassifier
from sklearn.ensemble import RandomForestClassifier
from sklearn.svm import SVC
from collections import Counter
from sklearn.model_selection import StratifiedKFold
import numpy as np

#from keras.wrappers.scikit_learn import KerasClassifier

#import matplotlib
import matplotlib.pyplot as plt
#import tensorflow as tf
from keras.models import Sequential
from keras.layers.core import Dense
from sklearn.feature_selection import RFE


class Ards_Pre(object):
 def __init__(self):

 self.raw_data_path = "./processed_data.xlsx"
 self.min_max_scaler = StandardScaler()
 Info_Dict = self.get_Info()
 print(Info_Dict.keys())
 for sheet_name, info in Info_Dict.items():
 if sheet_name =="单因素分析强相关指标":
 info = info[self.features]
 info = info.dropna()
 print(info.shape)
 y = info["是否有ARDS（有＝1，无＝0）"]
 pos_counts = Counter(y)[1]
 neg_counts = Counter(y)[0]
 neg_data = info[info["是否有ARDS（有＝1，无＝0）"] == 0]
 pos_data = info[info["是否有ARDS（有＝1，无＝0）"] == 1]
 #print(pos_data)
 neg_data_1 = neg_data.sample(n = pos_counts)
 # print("负样本数量", neg_data_1)
 print("负样本数量", neg_data_1.shape[0])
 data_info_1 = pd.concat([neg_data_1, pos_data], axis=0)
 print("用于模型的数据data_info_1", data_info_1.shape)
 self.X_train, self.X_val, self.y_train, self.y_val, self.X, self.y, self.features = self.data_split(
 data_info_1)
 self.fpr_DT, self.tpr_DT = self.Decison_Tree()
 self.fpr_RF, self.tpr_RF = self.RF_model()
 self.fpr_SVM, self.tpr_SVM = self.SVM()
 self.fpr_DNN, self.tpr_DNN = self.DNN_Model()
 self.fpr_LR, self.tpr_LR = self.LR_model()
 self.DNN_cross_validation()
 self.plot_ROC()


 def get_Info(self):
 *"""* ***:return****:
 """* sheet_info= dict()
 Info = pd.read_excel(self.raw_data_path,sheet_name=None)
 for sheet_name, info in Info.items():
 sheet_info.update({sheet_name:info})
 return sheet_info

 def plot_ROC(self):
 *"""
 1.绘制多条ROC曲线* ***:return****:
 """* #plt.figure()
 figure,ax = plt.subplots()
 lw = 2
 plt.figure(figsize=(10, 10))
 plt.plot(self.fpr_DT, self.tpr_DT, color='blue', lw=lw) ###假正率为横坐标，真正率为纵坐标做曲线

 plt.plot(self.fpr_RF, self.tpr_RF, color='black', lw=lw)

 plt.plot(self.fpr_LR, self.tpr_LR, color='red', lw=lw)

 plt.plot(self.fpr_SVM, self.tpr_SVM, color='dodgerblue', lw=lw)

 plt.plot(self.fpr_DNN,self.tpr_DNN, color = 'violet',lw=lw)

 #plt.xlim([0.0, 1.0])
 #plt.ylim([0.0, 1.05])
 ###设置刻度值字体大小
 plt.tick_params(labelsize=20)
 labels = ax.get_xticklabels() + ax.get_yticklabels()
 [label.set_fontname('Times New Roman') for label in labels]
 font2 = {'family': 'Times New Roman',
 'weight': 'normal',
 'size': 30,
 }
 plt.ylabel('Sensitivity',font2)
 plt.xlabel('1-Specificity',font2)

 plt.title('Receiver Operating Characteristic',font2)
 #plt.legend(loc="lower right")
 #plt.savefig(base + "roc_img\\sobel_roc.jpg")
 plt.show()


 def data_split(self,info_data):
 *"""
 1.划分测试集，验证集
 2.归一化处理* ***:return****:
 """* features = info_data.columns.tolist()
 #print(features)
 features.remove("是否有ARDS（有＝1，无＝0）")
 print("特征维度:",len(features))
 x = info_data[features]
 y = info_data["是否有ARDS（有＝1，无＝0）"]
 print(Counter(y))
 X_train, X_val, y_train, y_val = train_test_split(x, y, test_size=0.3, random_state=101)
 return X_train,X_val,y_train,y_val,x,y,features

 def Decison_Tree(self):
 *"""
 1.构建决策树模型
 2. 预测 ards 发生概率* ***:return****:
 """* decision_tree_model = DecisionTreeClassifier(random_state=101, criterion="entropy", max_leaf_nodes=5)
 #decision_tree_model.fit(self.X_train, self.y_train)
 #scores = cross_val_score(decision_tree_model,self.X_train,self.y_train,scoring="roc_auc",cv= 10)
 #print(scores.mean())
 #print(self.X_train.shape)
 #print(self.y_train.shape)
 ####使用十折交叉验证
 kfold = KFold(n_splits=10)
 param_grid = {"criterion": ["entropy"]}
 #decisionTree_model = DecisionTreeClassifier(random_state=101, criterion="entropy", max_leaf_nodes=50)

 grid = GridSearchCV(decision_tree_model, param_grid, cv=kfold)
 grid.fit(self.X_train, self.y_train.ravel())
 print(grid.cv_results_)
 #predict_tree = grid.best_estimator_.predict(self.X_val)
 #predict_score = grid.best_estimator_.predict_proba(self.X_val)
 #accuracy = accuracy_score(self.y_val, predict_tree)
 ########不使用十折交叉验证
 decision_tree_model.fit(self.X_train, self.y_train)
 predict_tree = decision_tree_model.predict(self.X_val)
 predict_score = decision_tree_model.predict_proba(self.X_val)
 accuracy = accuracy_score(self.y_val, predict_tree)
 print('DecisonTree model---Test accuracy: {:.4f}'.format(accuracy))
 auc = metrics.roc_auc_score(self.y_val, predict_score[:, 1])
 print('DecisonTree model---Test AUC: {:.4f}'.format(auc))
 print("输出验证集预测结果的混淆矩阵\n", confusion_matrix(self.y_val, predict_tree, labels=[0, 1])) # 输出预测结果的混淆矩阵
 print("-------------------------")
 print("验证集打印分类报告\n", classification_report(self.y_val, predict_tree)) # 打印分类报告
 print("-------------------------")
 fpr, tpr, thresholds = metrics.roc_curve(self.y_val, predict_score[:, 1], drop_intermediate=False)
 #fpr = 0
 #tpr = 0
 return fpr, tpr


 def RF_model(self):
 *"""
 1.构建随机森林模型
 2.预测ards 发生概率* ***:return****:
 """* print("............RandomForest Model................")
 print("训练集样本统计:", self.X_train.shape)

 random_forest_model = RandomForestClassifier(random_state=50, criterion="entropy", n_estimators=40)
 #random_forest_model.fit(self.X_train, self.y_train.ravel())
 #####十折交叉验证
 kfold = KFold(n_splits=10)
 param_grid = {"criterion": ["entropy"]}
 #decisionTree_model = DecisionTreeClassifier(random_state=101, criterion="entropy", max_leaf_nodes=50)
 grid = GridSearchCV(random_forest_model, param_grid, cv=kfold)
 grid.fit(self.X_train, self.y_train.ravel())
 print(grid.cv_results_)
 #print("-------测试集做模型验证结果--------")
 #importance = random_forest_model.feature_importances_
 #importance_dict = {"变量":self.features,"重要性":importance}
 #importance_df = pd.DataFrame(importance_dict)
 #importance_df.to_excel("/Users/sunnannan/Desktop/data_v1/RF_4_variables.xlsx")


 #predict_random_forest = grid.best_estimator_.predict(self.X_val)
 #predict_score = grid.best_estimator_.predict_proba(self.X_val)
 #########非交叉验证
 random_forest_model.fit(self.X_train, self.y_train.ravel())
 predict_random_forest = random_forest_model.predict(self.X_val)
 predict_score = random_forest_model.predict_proba(self.X_val)

 accuracy = accuracy_score(self.y_val, predict_random_forest)
 print('RandomForest model---Test accuracy: {:.4f}'.format(accuracy))
 print("输出验证集预测结果的混淆矩阵\n", confusion_matrix(self.y_val, predict_random_forest, labels=[0, 1])) # 输出预测结果的混淆矩阵
 print("-------------------------")
 print("验证集打印分类报告\n", classification_report(self.y_val, predict_random_forest)) # 打印分类报告
 print("-------------------------")
 auc = metrics.roc_auc_score(self.y_val, predict_score[:, 1])
 print('RandomForest model---Test AUC: {:.4f}'.format(auc))
 fpr, tpr, thresholds = metrics.roc_curve(self.y_val, predict_score[:, 1], drop_intermediate=False)
 return fpr, tpr


 def LR_model(self):
 *"""
 1.构建逻辑回归模型
 2.预测ards 发生概率* ***:return****:
 """* print("-------LR-model--------")
 lr_model = LogisticRegression(random_state=101, solver='liblinear')
 pipe = Pipeline([("scaler", self.min_max_scaler), ("logistic", lr_model)])
 ########10折交叉验证
 kfold = KFold(n_splits=10)
 param_grid = {"logistic__C": [1.0]}
 grid = GridSearchCV(pipe, param_grid, cv=kfold)
 grid.fit(self.X_train, self.y_train.ravel())
 print(grid.cv_results_)
 #pipe.fit(self.X_train,self.y_train)
 ######预测
 #predict_score = grid.best_estimator_.predict_proba(self.X_val)
 #y_pre = grid.best_estimator_.predict(self.X_val)
 # y_test_pre = pipe.predict(self.test)
 # y_test_score = pipe.predict_proba(self.X_test)
 # 输出分类错误样本
 # self.error_classfied_samples("logistic",y_pre)
 ######
 pipe.fit(self.X_train, self.y_train)
 predict_score = pipe.predict_proba(self.X_val)
 y_pre = pipe.predict(self.X_val)
 accuracy = accuracy_score(self.y_val, y_pre)
 # accuracy_test = accuracy_score(self.y_test, y_test_pre)
 print("-------------------------")
 print('LR model---Val accuracy: {:.4f}'.format(accuracy))
 print("-------------------------")
 print("输出验证集预测结果的混淆矩阵\n", confusion_matrix(self.y_val, y_pre, labels=[0, 1])) # 输出预测结果的混淆矩阵
 print("-------------------------")
 print("验证集打印分类报告\n", classification_report(self.y_val, y_pre)) # 打印分类报告
 print("-------------------------")
 auc = metrics.roc_auc_score(self.y_val, predict_score[:, 1])
 print("LR model-----AUC:",auc)
 fpr, tpr, thresholds = metrics.roc_curve(self.y_val, predict_score[:, 1], drop_intermediate=False)
 return fpr, tpr

 def SVM(self):
 *"""
 1.建立支持向量机模型
 2.预测ards 发生概率* ***:return****:
 """* print("训练集样本统计:", self.X_train.shape)
 svm_model = SVC(kernel='rbf', probability=True, C=10, gamma=0.001, decision_function_shape='ovr')
 #svm_model.fit(self.X_train, self.y_train.ravel())
 # print(svm_model.n_support_)
 #####十折交叉验证
 kfold = KFold(n_splits=10)
 param_grid = {"gamma": [0.001]}
 grid = GridSearchCV(svm_model, param_grid, cv=kfold)
 grid.fit(self.X_train, self.y_train.ravel())
 print(grid.cv_results_)
 #predict_svm = grid.best_estimator_.predict_proba(self.X_val)
 #accuracy = accuracy_score(self.y_val, predict_svm)
 #print('Test accuracy: {:.4f}'.format(accuracy))

 #print("输出验证集预测结果的混淆矩阵\n", confusion_matrix(self.y_val, predict_svm, labels=[0, 1])) # 输出预测结果的混淆矩阵
 #print("-------------------------")
 #print("验证集打印分类报告\n", classification_report(self.y_val, predict_svm)) # 打印分类报告
 #print("-------------------------")
 svm_model.fit(self.X_train, self.y_train.ravel())
 predict_svm = svm_model.predict_proba(self.X_val)
 auc = metrics.roc_auc_score(self.y_val, predict_svm[:, 1])


 print('SVM model---Test AUC: {:.4f}'.format(auc))
 #fpr = 0
 #tpr = 0
 fpr, tpr, thresholds = metrics.roc_curve(self.y_val, predict_svm[:, 1], drop_intermediate=False)
 #accuracy = accuracy_score(self.y_val, predict_svm)
 #print('Test accuracy: {:.4f}'.format(accuracy))
 #print("输出验证集预测结果的混淆矩阵\n", confusion_matrix(self.y_val, predict_svm, labels=[0, 1]))
 #print("-------------------------")
 #print("验证集打印分类报告\n", classification_report(self.y_val, predict_svm))
 print("-------------------------")
 return fpr, tpr
 def get_fc_model(self):
 model = Sequential();
 model.add(Dense(64, input_shape=(20,), activation='relu'))
 model.add(Dense(32, activation='relu'))
 #model.add(Dense(16, activation='relu'))
 model.add(Dense(8, activation='relu'))
 model.add(Dense(1, activation='sigmoid'))
 model.compile(optimizer='Adam', loss='mean_squared_error',
 metrics=['accuracy'])
 model.optimizer.lr = 0.01

 return model

 def DNN_Model(self):
 *"""
 1.建立DNN模型
 2.模型预测
 3.输出结果* ***:return****:
 """* fc_model = self.get_fc_model()
 #fc_model.compile(optimizer='Adam', loss='mean_squared_error',
 # metrics=['accuracy'])
 #fc_model.optimizer.lr = 0.01
 #print(self.X_train.values,self.y_train.values)
 fc_model.fit(self.X_train.values, self.y_train.values, epochs=100,batch_size=16)
 predictions = fc_model.predict_proba(self.X_val, verbose=0)
 y_pre = fc_model.predict_classes(self.X_val,verbose=0)
 #print(len(predictions))
 #predictions = predictions.reshape(1, 183)
 #predictions = predictions[0]
 #print(predictions)
 fpr_keras, tpr_keras, thresholds_keras = metrics.roc_curve(self.y_val, predictions,drop_intermediate = False)
 #print(fpr_keras,tpr_keras,thresholds_keras)
 # 计算 AUC
 AUC = metrics.auc(fpr_keras, tpr_keras)

 print("DNN Model-AUC",AUC)
 print("DNN Model 输出验证集预测结果的混淆矩阵\n", confusion_matrix(self.y_val, y_pre, labels=[0, 1]))
 #tpr_keras =0
 #fpr_keras = 0
 return fpr_keras,tpr_keras
 def DNN_cross_validation(self):
 *"""* ***:return****:
 """* seed = 4
 kfold = StratifiedKFold(n_splits=10, random_state=seed, shuffle=True)
 cvscores = []

 for train, validation in kfold.split(self.X_train.values, self.y_train.values):
 # model
 fc_model = self.get_fc_model()
 # train
 fc_model.fit(
 self.X_train.values[train], self.y_train.values[train],
 epochs=100,
 batch_size=32,
 verbose=0,
 )
 #predictions = fc_model.predict_proba(self.X_train.values[validation], verbose=0)
 #fpr_keras, tpr_keras, thresholds_keras = metrics.roc_curve(self.y_train.values[validation], predictions, drop_intermediate=False)
 #print(fpr_keras,tpr_keras,thresholds_keras)
 # 计算 AUC
 #AUC = metrics.auc(fpr_keras, tpr_keras)

 #print("DNN Model-AUC", AUC)
 scores = fc_model.evaluate(self.X_train.values[validation], self.y_train.values[validation], verbose=0)
 print('%s:%.2f%%' % (fc_model.metrics_names[1], scores[1]*100))
 cvscores.append(scores[1]*100)
 print('%.3f (+/- %.3f)' % (np.mean(cvscores), np.std(cvscores)))


if __name__ == "__main__":
 Ards_Pre = Ards_Pre()
