# Supplementary figures and images for: Risk factors analysis of COVID-19 patients with ARDS and prediction based on machine learning
Source: Sci Rep. 2021 Feb 3;11:2933. doi: 10.1038/s41598-021-82492-x (PMC7858607; doi:10.1038/s41598-021-82492-x)

**Figure Legends**

**Figure S1** Deep Learning (DL) algorithm framework

**Figure S1**


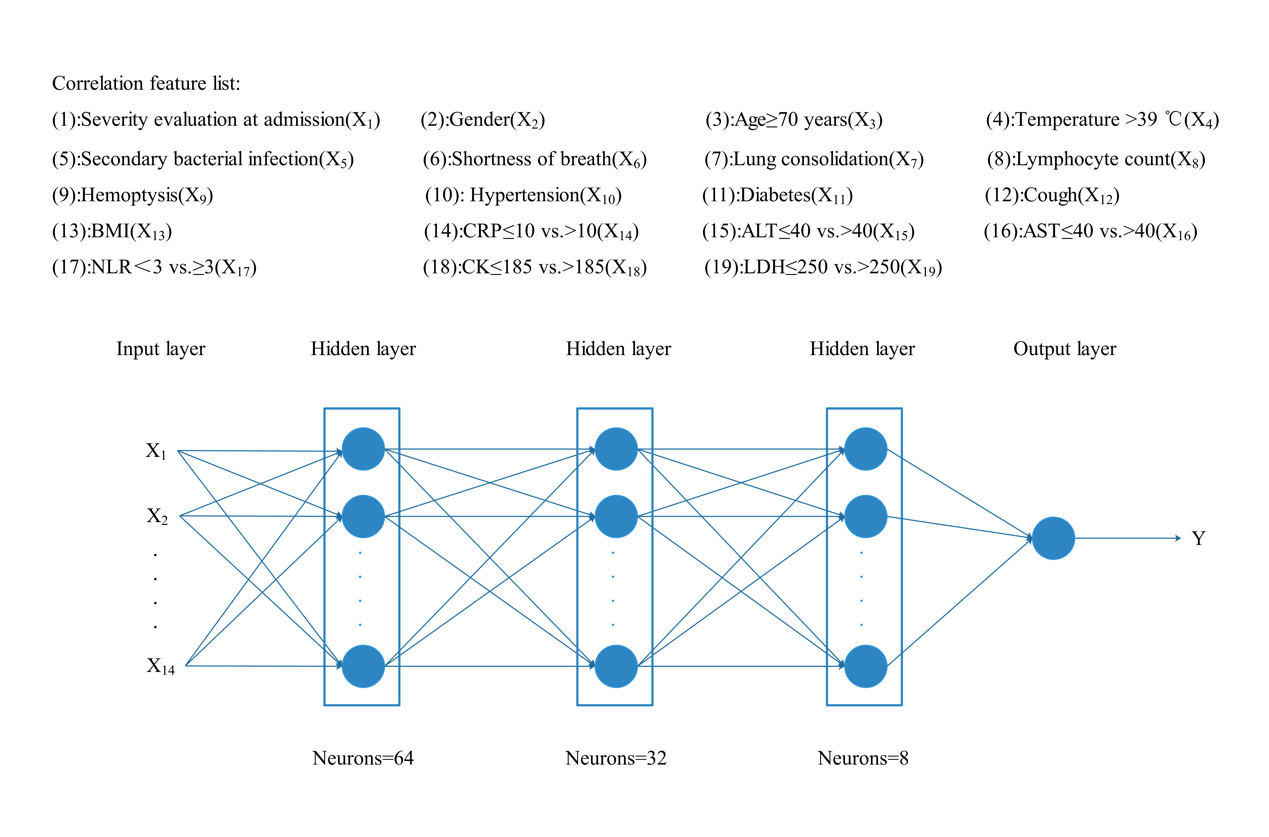

Supplement: Supplementary file 3 — Supplementary Information 3. [file 41598_2021_82492_MOESM3_ESM.docx]
